# Do social media interventions increase vaccine uptake?

**DOI:** 10.3389/fpubh.2023.1077953

**Published:** 2023-06-29

**Authors:** Francesca Maria Grosso, Maria Elisabetta Baldassarre, Roberto Grosso, Federica Di Mauro, Chiara Greco, Silvia Greco, Nicola Laforgia, Antonio Di Mauro

**Affiliations:** ^1^Department of Biomedical Sciences for Health, Postgraduate School of Public Health, University of Milan, Milan, Italy; ^2^Department of Biomedical Science and Human Oncology, Neonatal Intensive Care Unit, Aldo Moro University of Bari, Bari, Italy; ^3^Pediatric Primary Care, National Pediatric Health Care System, Bari, Italy; ^4^Department of Prevention, Local Health Authority of Bari, Bari, Italy; ^5^Department of Biomedical Science and Human Oncology, Hygiene and Preventive Medicine (Public Health), Aldo Moro University of Bari, Bari, Italy; ^6^Department of Pediatrics, Gabriele d'Annunzio University of Chieti and Pescara, Chieti, Italy; ^7^Pediatric Primary Care, National Pediatric Health Care System, Margherita di Savoia, Barletta-Andria-Trani, Italy

**Keywords:** vaccine hesitancy, COVID-19, social media, vaccine campaign, primary care setting

## Abstract

**Introduction:**

The Italian mass COVID-19 vaccination campaign has included children aged 5–11 years as part of the target population since December 2021. One of the biggest challenges to vaccine uptake was vaccine hesitancy among parents and children's caregivers. Primary care pediatricians (PCPs), as the first point of contact between the National Health Service (NHS) and parents/caretakers, initiated various communication strategies to tackle this hesitancy. This study aims to evaluate the impact of a PCP-led social media intervention and a digital reminder service (DRS) on parental hesitancy regarding vaccinating their 5–11-year-old children against COVID-19.

**Methods:**

A prospective cohort study was designed, and the chosen target populations were parents and caretakers of children aged 5–11 years. Two PCP cohorts were recruited. The first group received a social media intervention and a DRS; while the second group did not. Both cohorts had access to traditional face-to-face and telephone-based counseling. The vaccination coverage rate in the two groups was evaluated.

**Results:**

A total of 600 children were enrolled. The exposed cohort (277 patients) received social media intervention, DRS, and counseling options (face-to-face and telephone-based), whereas the non-exposed cohort (323 patients) received only counseling options. In total, 89 patients from the exposed cohort did not receive any dose of the COVID-19 vaccine (32.5%), 165 were fully immunized (59.5%), and 23 received only one dose (8.5%). A total of 150 non-exposed patients did not receive any dose of the COVID-19 vaccine (47%), 147 were fully immunized (45.5%), and 24 only received one dose (7.4%). The difference between the two groups was statistically significant (chi square = 11.5016; *p* = 0.0006).

**Conclusion:**

Social media and DRS interventions had a positive impact on vaccine uptake and may be helpful in tackling vaccine hesitancy. Better-designed studies are needed to corroborate these findings.

## 1. Introduction

The Italian universal COVID-19 vaccination campaign was launched in December 2020, targeting adults and older people. After 1 year, vaccination was also extended to the pediatric population of 5–11 years of age. However, shortly after the authorization granted by the Food and Drug Administration and the European Medicines Agency, a wave of vaccine hesitancy arose nationwide: vaccine hesitancy refers to the delay in accepting or refusing vaccinations (1), despite the availability of vaccination services (2) and is a potential threat to coverage. In Apulia, a southern region of Italy, COVID-19 vaccine pediatric hubs were set up in various locations (e.g., schools and gyms) around Apulian cities. Primary care pediatricians (PCPs) were in charge of delivering the vaccine. According to the national guidelines, the regional Apulian government offered two 10 μg doses of the Comirnaty vaccine administered 21 days apart, free of charge, to children aged 5–11 years.

Extensive research has been conducted on the importance of primary care doctors in increasing vaccine acceptance. Recent studies reveal that these family physicians are a trusted source of information and play a vital role in addressing vaccine hesitancy (3). Being the first point of contact with the National Health System for most individuals, general practitioners and PCPs bring healthcare closer to the public. According to the Alma-Ata declaration in 1978 and various studies (4), primary care is the critical link to a flourishing healthcare system. There is a proven relationship between robust primary care and better population health outcomes (5). Multiple authors have emphasized, for example, the importance of primary care in increasing vaccine uptake. However, despite this knowledge, there are still barriers to achieving this goal; for example, combining research and good clinical practice in primary care (6). Research shows that effective communication is essential in increasing vaccine acceptance among parents who are hesitant (7–9). Physicians are critical in providing information and support to address vaccine safety and effectiveness concerns, as they are often asked about these issues. Communication strategies can take various forms, including traditional one-on-one counseling and utilizing social media and instant messaging as new communication channels.

The use of social media as a source of information has increased during the COVID-19 pandemic. However, there is a need for further research to study its impact on physician–patient communication, particularly in primary care settings (10).

The purpose of this study is to examine how social media intervention and digital reminder service (DRS) impact the rate of COVID-19 vaccination uptake among children aged between 5 and 11 years in a primary care setting. In addition, the study compares the results of using these services to the results of not using them.

## 2. Materials and methods

We have conducted a longitudinal cohort study for 4 months, from 16 December 2021 to 30 March 2022, in two pediatric primary care offices (PPCOs) in the Apulia region. This study aimed to evaluate the effectiveness of social media-based intervention and DRS compared to no organized digital intervention. Both groups were given access to traditional in-person or remote vaccination-related counseling during working hours if requested. The intervention was implemented at the PPCO in Margherita di Savoia (ASL BAT), while the other PPCO in Palese (ASL BA) served as the control group. The study included all children aged between 5 and 11 years, who were enrolled at the two PPCOs, at the start of the research, as per the regulations of the local health authority. The catchment area of each PPCO was defined based on its geographical location.

This study focused on children whose parents received social media-based vaccine education interventions to address their concerns about the COVID-19 vaccine. Additionally, they received digital appointment reminders. The social media interventions were created and/or mediated by their PCP and shared through a professional Facebook page (https://www.facebook.com/antoniodimauropediatra) with over 50,000 followers. The page regularly featured posts with reliable vaccine information, including infographics, videos from trusted sources, and Q&A sessions with experts, e.g., the Italian Society of Pediatrics (https://www.facebook.com/societaitalianadipediatria). Other Facebook posts were arranged into short, easy-to-read paragraphs, discussing the risks and benefits of vaccines and news on pediatric COVID-19 and its management. The pediatrician in charge of the ASL BAT PPCO certified the validity and trustworthiness of the content.^1^ During the study period, a total of 102 posts were published on Facebook. These posts received 462,883 interactive visualizations, 37,915 likes, 5,740 shares, and 3,481 comments. It is estimated that the social network activity reached 1,488,437 Facebook users. This data were extracted through Facebook Insight. Additionally, four active digital messages were sent to parents/caretakers to remind them to vaccinate children on dedicated open days through messaging services, Pediatotem and Whatsapp, as part of the DRS program.

The control group received no specific communication through social media. However, they could receive counseling from their PCP in person or remotely during working hours, if requested. Due to the COVID-19 pandemic, PCPs only saw patients by appointment and did not accept walk-ins.

With an alfa level of 0.05 and a power of 95% to detect an absolute difference of 30% between the coverage of cases and controls, a sample size of 235 in each group was calculated, assuming that 18% of the age group had obtained at least one dose of vaccine, as reported by the US administration in December 2021. The lists of children aged 5–11 years from the two cohorts were obtained from the regional database (EDOTTO), which stores databases of PPCO-registered patients. We acquired information on the administration dates of the first and second COVID-19 vaccine doses from the Apulian vaccination registry (GIAVA) for both groups. However, some of the data were missing or only partially available. Unfortunately, we were also unable to access individual-level age data due to aggregation. In addition, data on sex were missing, so we had to rely on names to retrieve individual-level data, which may have introduced some inaccuracies. To connect the data, we used Microsoft Access to link it through a primary key. All data were anonymized for privacy purposes. We only included children who had received two vaccine doses when calculating the coverage. Those who had received only one dose were not included. The data were collected on 30 March 2022 and analyzed using SPSS 28 software. We compared the percentage of fully vaccinated children in the exposed group to that of the non-exposed group. We used the Pearson chi-square test to determine whether the difference was significant. A *p* < 0.05 was considered statistically significant.

## 3. Results

A total of 600 patients aged 5–11 years were included in the study. In total, 277 patients were registered to the PPCO of Margherita di Savoia (exposed cohort), of which 49% were boys and 51% were girls.

**Figure 1:**
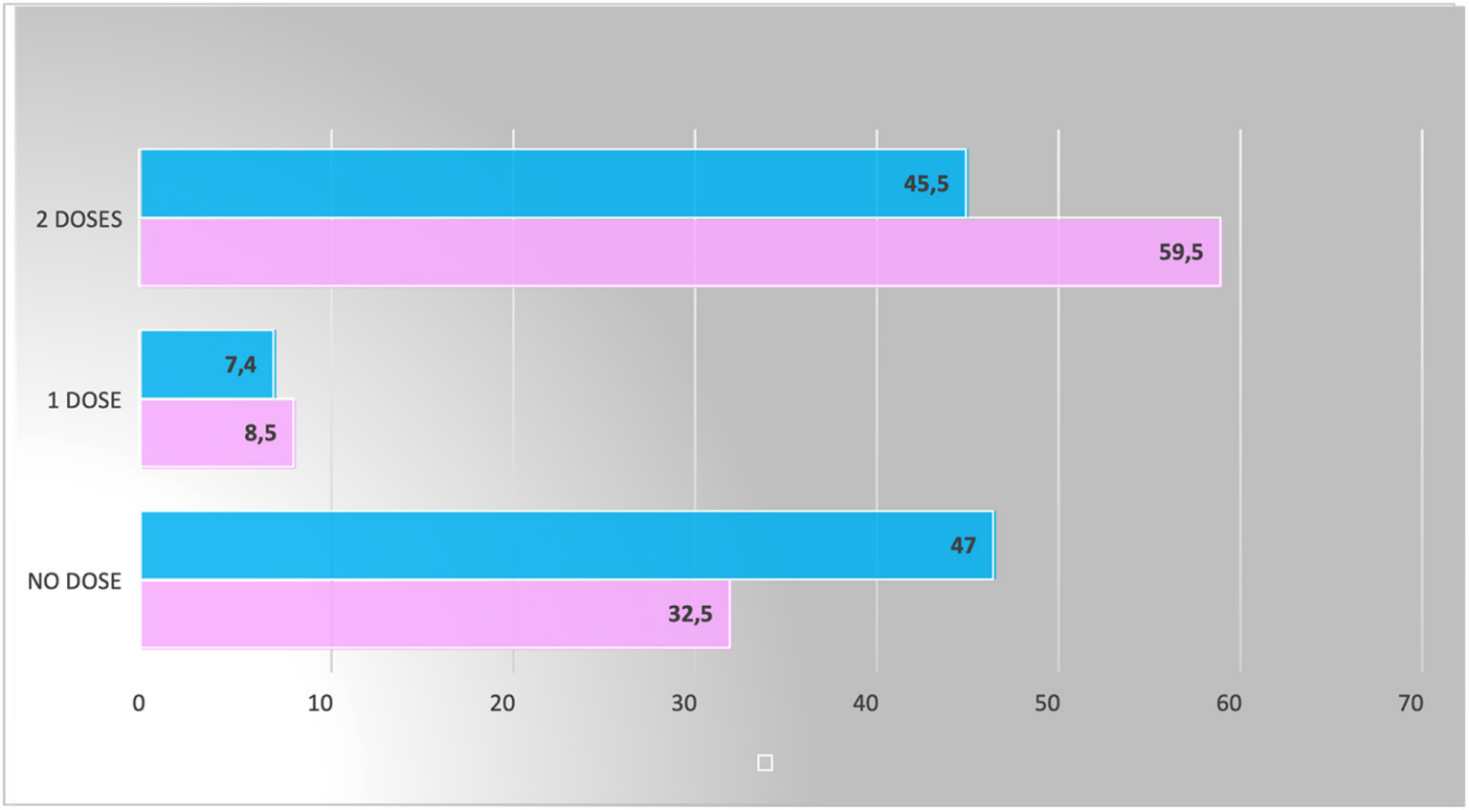
Proportions of fully immunized, partly immunized, and non-immunized children compared between the two cohorts (The pink color represents the exposed group, and the blue color represents the unexposed).

In this cohort, 89 patients did not receive any dose of the COVID-19 vaccine (32.5%), 165 were fully immunized (59.5%), and 23 received only one dose (8.5%). The total number of children aged 5–11 years registered to the Palese PPCO (non-exposed cohort) was 323, of which 45% were girls and 55% were boys. In total, 152 did not receive any dose of the COVID-19 vaccine (47%), 147 were fully immunized (45.5%), and 24 only received one dose (7.4%). The results are shown in Figure 1.

The proportion of children fully immunized in the exposed cohort was 59.5%, while in the non-exposed cohort, only 45.5% were fully vaccinated. The proportion of the difference between the two groups was 14%, and there is strong evidence that this difference might not be due to chance (chi square = 11.5016; *p* = 0.0006). The odds ratio calculation yielded a value of 1.8 (95% CI: 1.2; 2.5), suggesting that parents exposed to this intervention are 80% more likely to vaccinate their children. However, confidence intervals are wide, indicating a significant uncertainty in the estimate.

## 4. Discussion

Numerous studies have highlighted the negative impact of social media as a source of fake news, misinformation, and conspiracy theories. Research has demonstrated that exposure to vaccine-critical content can decrease the intention to vaccinate. Specifically, users who are exposed to vaccine-critical content for 5–10 min a day are more worried about the potential risks of vaccination compared to those who view evidence-based medical content (11–13).

Many individuals within the healthcare community believe that social media can be an effective tool for disseminating scientific information to a wider audience. Regrettably, this potential benefit appears to be undermined by the growing number of people who use these platforms to promote vaccine hesitancy (14, 15). A thorough review of research studies investigating the connection between social media and COVID-19 vaccination indicates that social media has an overall negative impact on people's willingness to get vaccinated. This study also reveals, however, that vaccine acceptance rates differ depending on which social media platform people use, suggesting that exposure to different types of content might influence vaccine hesitancy (16). While the relationship between social media and vaccine hesitancy is complex and multifaceted, these studies highlight the urgent need for PCPs, other health managers, and healthcare providers to actively work to combat misinformation and promote accurate information about vaccines on social media.

Parents play an essential role in pediatric vaccination uptake and should receive adequate support and information from their healthcare providers, who are highly qualified people to address their concerns. The Center for Disease Control and Prevention (CDC) released guidance on vaccine protection that highlights the importance of parents feeling cared for by physicians who lead by example and can provide both personal stories and scientific facts while taking the time to listen during consultations (17). Without proper guidance, parents may turn to the internet for information about vaccines, which can be risky as they may come across misleading content. Research has suggested that parents who actively seek vaccine information online tend to have more concerns about vaccine safety, effectiveness, and disease susceptibility than those who do not use them (18). According to a study, social groups that had physicians as influencers on social media were more likely to accept vaccines. This suggests a growing need for a “public physician” role on social media, where a physician can represent and share information with the public (16).

Literature studies describing and analyzing the impact of health system interventions on vaccine uptake are scarce, inconclusive, and often not well-targeted: one systematic review discovered that the majority of studies were predominantly focused on individuals with higher levels of education. However, these studies failed to take into account the potential impact of language and cultural differences, which may also contribute to vaccine hesitancy (19). A systematic review by Kaufman et al. shows that intervention could increase early vaccine adherence in populations lacking an understanding of the role of the vaccine. At the same time, their impact is less evident in people whose primary barrier is vaccine hesitancy (20). Primary care settings are potentially valuable places to test the effectiveness of health education interventions (21). Unfortunately, these settings often lack the necessary resources to conduct such studies.

This study has several limitations. First, we could not determine whether the positive impact on vaccination was due to the social media intervention or the digital reminders received solely by the intervention group. While there is existing literature supporting the effectiveness of digital reminders in increasing vaccination rates in children aged 0–5 years and 11–18 years, the data available are scarce for the 5–11 years age group (22). Therefore, we cannot rule out the possibility that the digital reminder was the main factor in boosting vaccination rates, rather than the social media intervention.

The data used in the study were obtained from regional software that only provided information on sex and age, making it difficult to compare the two cohorts at the beginning of the study. There was also no record of any face-to-face or remote counseling that may have taken place. Differences in socioeconomic status, age, gender, and parental attitudes toward vaccination could have influenced the results. To reduce any potential bias, collecting primary data would be beneficial. In addition, exposure to Facebook posts could not be assessed precisely; other social media influencers or web pages might have impacted the willingness to uptake vaccination. Off-line influences were not evaluated, but they are likely to account for a more or less positive impact on the cohorts. Our study was conducted in a region with a highly effective vaccination protocol, so it may only be relevant to certain areas. To confirm our findings, more randomized controlled trials need to be performed. However, our study is valuable because it provides some useful, albeit somewhat confounded evidence on the impact of social media educational interventions in primary care settings.

## 5. Conclusion

Primary care-mediated social media has the potential to be an effective tool for implementing public health. It can build on PCP trust and reach many patients simultaneously, transcending space and time. Additionally, it tends to provide information in accessible and understandable ways, which can enhance health literacy, ownership, and utility of end users. Although this study shows that social media interventions combined with DRS may increase vaccine uptake, we cannot definitively conclude that they effectively address parental vaccine hesitancy due to the study's limitations. Further research is necessary to fully understand the relationship between social media exposure and vaccination uptake. Additional efforts and resources should be dedicated to exploring this association. To combat vaccine hesitancy and improve vaccination coverage, we call for more ongoing scientific partnerships between universities, local health organizations, and PCPs to develop such innovative solutions.

## Data availability statement

The raw data supporting the conclusions of this article will be made available by the authors, without undue reservation.

## Ethics statement

Ethical review and approval was not required for the study on human participants in accordance with the local legislation and institutional requirements. Written informed consent to participate in this study was provided by the participants' legal guardian/next of kin.

## Author contributions

AD planned the study, coordinated the study, and carried out the social media intervention. RG carried out the traditional face-to-face counseling. FG performed statistical analysis of data and wrote the first draft of the manuscript. FD, MB, SG, and CG explored the literature. NL revised the final manuscript. All authors have read and approved the final manuscript.

## Footnotes

^1^Posts used in this study are easily accessible and manageable to allow replication studies in previously cited Facebook Pages.
